# Some of the Great Bridges

**Published:** 1881-08

**Authors:** 


### SOME OF THE GREAT BRIDGES.

Robert Stephenson, great engineer as he was, reported that suspension bridges would never do for steam. John A. Roebling answered with the Niagara Suspension Bridge, the cheapest structure and one of the best ever built for such a necessity.

In Menai Strait, which divides an island from the northwestern corner of Wales, the tide rises to the height of 30 feet sometimes, and generally 12 feet. The British Government erected a bridge on the great high road from England to Ireland over this strait in 1826. It is a suspension bridge, built by Telford, on chains, and cost $600,000 (gold) at that time. It is 100 feet above water. Twenty years afterward George Stephenson began to build the tubular bridge three miles above, spanning the same strait. It took five years, and trains crossed it in 1850. It has four spans, the two in the middle being 460 feet wide each, and the whole bridge is about 1,840 feet long. It is 123 feet above high-water-mark, and cost $3,000,000.

The Niagara suspension bridge, built by Roebling in 1852, cost only $500,000, is 800 feet long, 230 feet above the river, and its towers are about 84 feet high. The Niagara foot-bridge, built in 1869, cost $175,000, and was said to be, when opened, the longest suspension bridge in the world, or 1,268 feet between towers.

The Cincinnati suspension bridge, by Roebling, stands next to the East River bridge, and is 1,057 feet between towers and 2,252 feet between the ends; the bridge is 103 feet above low water, the towers are 230 feet high, and each is taller and larger than the Bunker Hill monument, and the structure cost $1,800,000 ; it was built by a company, and charges three cents toll per man. This bridge has been in most useful operation since about 1867; it was eleven years between its commencement and opening.

Roebling, the projector of the Brooklyn Bridge, was the greatest bridge-builder in the world. He started the making of wire cordage in America, and built suspension bridges to carry the aqueducts of canals across rivers, and engineered the Pennsylvania Railroad across the mountains. The Brooklyn Bridge, between towers, is 1,595 feet long. Behind the towers there are 940 feet each side, back to the anchorages. The whole length of the bridge and approaches is 6,000 feet. It is one of the widest bridges in the world, 85 feet, with a promenade 13 feet wide, two railroad tracks and four carriage and two horse-car tracks. It is 135 feet in the centre above the water. The rock on which the towers rest is about ninety feet below the surface of the water on the New York side, and half that depth on the Brooklyn side—the most stupendous thing about the structure. Each tower is 134 feet long by 56 wide, and at the top these dimensions are reduced to 120 feet by forty, or the size of a very large house. Each tower is 268 feet above high water. It is 1,336 feet from the beginning of the causeway on Chatham street out to the anchorage on the New York shore. The architect of the bridge received his death wound almost at its inception.

